# Trigger-Free Neuraxial Anaesthesia for Emergency Evacuation of Retained Products of Conception in Limb-Girdle Muscular Dystrophy: A Case Report and Literature Review

**DOI:** 10.7759/cureus.95759

**Published:** 2025-10-30

**Authors:** Reem AlAamer, Ahmed Alanzi, Dawood Alatefi, Abdulrahman A Alselaiti

**Affiliations:** 1 Anesthesia and Pain Management, King Hamad University Hospital, Muharraq, BHR; 2 Neurological Surgery, University of Jordan, Amman, JOR

**Keywords:** evacuation of retained products of conception, limb-girdle muscular dystrophy, neuraxial anaesthesia, obstetric and gynaecologic surgery, spinal anaesthesia, trigger-free anaesthesia

## Abstract

Limb-girdle muscular dystrophy (LGMD) carries anaesthetic risks including anaesthesia-related rhabdomyolysis, hyperkalaemia, and prolonged neuromuscular blockade. Trigger-free techniques that avoid volatile agents and depolarising neuromuscular blockers (NMBs) are preferred.

A 31-year-old woman with LGMD presented four days after a second-trimester miscarriage with pain and tissue passage requiring emergency evacuation of retained products of conception (ERPC). Examination showed proximal limb weakness without cardiopulmonary compromise; routine laboratory results were normal. A vapour-free, clean anaesthesia workstation was prepared and succinylcholine/volatile agents strictly avoided. Single-shot spinal anaesthesia (0.5% hyperbaric bupivacaine 10 mg at L3-4) provided adequate surgical conditions. Intraoperative haemodynamics were stable; no sedatives or NMBs were used. Surgery lasted ~20 minutes. Recovery in the post-anaesthesia care unit (PACU) was uncomplicated with an Aldrete score of 10 and same-day discharge.

For urgent gynaecological surgery in LGMD, neuraxial anaesthesia can provide reliable anaesthesia while avoiding recognised triggers of rhabdomyolysis and hyperkalaemia. Meticulous preoperative planning (vapour-free machine, avoidance of depolarising agents, and ready total intravenous anaesthesia (TIVA) backup) supports safety in this population.

## Introduction

Limb-girdle muscular dystrophy (LGMD) encompasses a heterogeneous group of inherited myopathies characterized by progressive weakness of the shoulder and pelvic girdle musculature, with variable cardiac and respiratory involvement [1]. Recent global estimates suggest that the prevalence of LGMD ranges between 1 in 14,000 and 1 in 123,000 individuals, with autosomal-recessive subtypes being more frequent in consanguineous populations and regional variations reported across Europe, the Middle East, and Asia [2].

These multisystem features create distinct perioperative risks, namely, anaesthesia-related rhabdomyolysis, hyperkalaemia, malignant-hyperthermia-like crises, and unpredictable responses to neuromuscular blockers (NMBs). Consequently, contemporary guidance favours trigger-free strategies that avoid volatile anaesthetic agents and succinylcholine (sux), coupled with vigilant monitoring and readiness for postoperative respiratory support when indicated [3-6].

While obstetric anaesthetic considerations in muscular dystrophies are described, specific guidance for urgent gynaecological procedures such as evacuation of retained products of conception (ERPC) is limited. Case reports describe both safe trigger-free total intravenous anaesthesia (TIVA) and successful neuraxial approaches for lower-abdominal and obstetric surgery in patients with LGMD [7]. Neuraxial anaesthesia offers theoretical advantages in this context by providing dense surgical anaesthesia without exposure to volatile agents or muscle relaxants, and by facilitating early recovery and discharge in appropriately selected patients [5,8,9].

We report the successful use of single-shot spinal anaesthesia for emergency ERPC in a woman with LGMD, implemented within a rigorously trigger-free pathway that included vapour-free machine preparation, strict avoidance of depolarizing agents and volatile anaesthetics, and a fully prepared TIVA contingency.

## Case presentation

A 31-year-old woman with known LGMD presented four days after a second-trimester miscarriage with lower-abdominal pain and intermittent tissue passage. She had no history of malignant-hyperthermia-like events, anaesthetic complications, or chronic cardiopulmonary disease. She was not taking statins and had no known drug allergies.

Examination revealed symmetric proximal limb weakness with preserved distal strength (Table 1). Airway evaluation was unremarkable. Vital signs were stable (Table 2). Baseline laboratory testing was unremarkable: full blood count (FBC) and serum biochemistry were within institutional reference intervals; serum potassium was normal; creatine kinase (CK) was not elevated; and hepatic function tests were within range (Table 3). A 12-lead electrocardiogram (ECG) showed sinus rhythm. Recent echocardiography demonstrated normal biventricular size and function. There were no symptoms suggestive of nocturnal hypoventilation; if available, spirometry was within acceptable limits for day-case surgery.

**Table 1:** Neurological examination summary MRC: Medical Research Council scale (0 = no contraction; 5 = normal strength; “4−” indicates slight reduction within grade 4).

Domain	Findings
Cranial nerves	Intact
Cognition	Preserved cognitive function
Sensation	Normal across modalities
Reflexes	Deep tendon reflexes sluggish
Muscle strength (MRC)	Pattern: Proximal > distal weakness, more pronounced in lower limbs
	Upper limbs: Proximal 3/5; elbow flexion 4−/5; hand grip ≈ 90%
	Lower limbs: Hip flexion/extension 3/5; right knee extension 3/5; left knee extension 2/5; hamstrings 3/5; ankle dorsiflexion 3/5; ankle plantarflexion 3/5

**Table 2:** Baseline vital signs HR: Heart rate; BP: Blood pressure; RR: Respiratory rate; SpO_2_: Oxygen saturation

Parameter	Value
HR	84 bpm
BP	118/72 mmHg
RR	16 breaths/min
SpO₂	100%
Temperature	36.8°C

**Table 3:** Baseline laboratory investigations FBC: Full blood count; CK: Creatine kinase; CRP: C-reactive protein; INR: International normalized ratio; aPTT: Activated partial thromboplastin time; ALP: Alkaline phosphatase; eGFR: Estimated glomerular filtration rate; ALT: Alanine aminotransferase; AST: Aspartate aminotransferase

Test	Result (units)	Reference range (adult)
FBC		
Haemoglobin	135 g/L	115–165 g/L
White cell count	7.0 ×10⁹/L	3.6–11.0 ×10⁹/L
Platelets	250 ×10⁹/L	140–400 ×10⁹/L
Electrolytes/Renal		
Sodium	140 mmol/L	133–146 mmol/L
Potassium	4.3 mmol/L	3.5–5.3 mmol/L
Chloride	102 mmol/L	95–108 mmol/L
Bicarbonate (HCO_3_^-^)	25 mmol/L	22–28 mmol/L
Urea	5.0 mmol/L	2.5–7.8 mmol/L
Creatinine	65 µmol/L	45–84 µmol/L
eGFR	100 mL/min/1.73 m²	≥90 (interpret with age/sex)
Liver function		
ALT	22 U/L	10–35 U/L
AST	24 U/L	15–34 U/L
ALP	75 U/L	30–130 U/L
Bilirubin (total)	12 µmol/L	0–21 µmol/L
Albumin	42 g/L	35–50 g/L
Muscle/Inflammatory		
CK	80 U/L	25–200 U/L
CRP	2 mg/L	0–5 mg/L
Glucose		
Fasting glucose	4.8 mmol/L	3.0–6.0 mmol/L
Random glucose	5.6 mmol/L	3.0–7.7 mmol/L
Coagulation		
INR	1.0	0.8–1.2
aPTT	30 s	25–37 s

Given the risks of anaesthesia-related rhabdomyolysis, hyperkalaemia, and altered responses to NMBs in LGMD, a trigger-free pathway was chosen. The anaesthesia workstation was thoroughly flushed to eliminate residual volatile agents, and all vapourisers were removed to ensure a vapour-free setup. Dantrolene availability and an malignant hyperthermia (MH)/rhabdomyolysis response checklist were verified. Succinylcholine and volatile agents were avoided. A TIVA backup was prepared should neuraxial anaesthesia prove insufficient. Standard monitoring with continuous temperature monitoring was used.

With standard monitors applied, single-shot spinal anaesthesia was performed at the L3-4 interspace using a pencil-point needle. After confirming free flow of cerebrospinal fluid aspiration, 0.5% hyperbaric bupivacaine 10 mg was administered intrathecally, achieving a T10 sensory level within minutes and a Bromage 2 motor block. No sedatives or NMBs were given. Prophylactic antibiotics were administered according to institutional policy, and oxytocin was started per obstetric guidance. Haemodynamics remained stable. Active warming and careful positioning were maintained. The procedure lasted about 20 minutes with minimal blood loss. No arrhythmias, hypercapnia, masseter spasm, or temperature rise occurred.

Recovery in the post-anaesthesia care unit (PACU) was uneventful. Analgesia was managed with paracetamol and an opioid-sparing regimen. There was no myalgia, dark urine, or other features of rhabdomyolysis. Postoperative electrolytes and CK were normal. The Aldrete score reached 10, and the patient was discharged the same day with instructions on hydration, warning signs, and follow-up. At two weeks, she remained well without complications.

## Discussion

Neuromuscular diseases (NMDs) encompass conditions that impair muscle function and demand specific peri-operative strategies to mitigate respiratory and cardiac risk [4]. Within this spectrum, LGMD represents a heterogeneous group of myopathies characterised by progressive proximal weakness with variable cardiopulmonary involvement and genetic diversity across autosomal-dominant and autosomal-recessive subtypes [1]. Anaesthetic hazards in LGMD cluster around triggered rhabdomyolysis and hyperkalaemia, events that may present with malignant-hyperthermia-like physiology, together with unpredictable responses to NMBs [3,5]. In line with these risks, contemporary guidance recommends trigger-free techniques that avoid volatile anaesthetics and succinylcholine, coupled with vigilant monitoring and readiness for postoperative respiratory support when indicated [2].

When feasible, regional anaesthesia is attractive because it avoids recognised pharmacological triggers and helps preserve spontaneous ventilation. Case-based evidence supports neuraxial strategies for lower-abdominal and gynaecological surgery in LGMD, including hysterectomy under regional anaesthesia with uneventful recovery, emergency caesarean delivery managed with combined spinal-epidural (CSE), spinal anaesthesia for caesarean section, obstetric management favouring neuraxial techniques in LGMD2I with cardiomyopathy, and normal vaginal delivery with careful monitoring where neuraxial was feasible [7-11]. Beyond obstetrics, abdominal wall surgery has also been performed successfully under CSE in LGMD, further reinforcing the practicality of regional approaches when not contraindicated [12]. Where regional is unsuitable or fails, propofol-based TIVA without volatiles or depolarising agents has been reported as a safe alternative in LGMD surgical cases, using minimal or no neuromuscular blockade and careful ventilation [3].

Our patient required urgent ERPC, a short but painful procedure in which rapid, reliable anaesthesia, haemodynamic stability, and preservation of spontaneous ventilation are desirable. Single-shot spinal anaesthesia provided dense surgical anaesthesia without sedatives or muscle relaxants, minimising cardiorespiratory disturbance and circumventing recognised triggers. Safety was enhanced by a rigorously trigger-free pathway: vapour-free machine preparation, avoidance of succinylcholine and volatile agents, continuous temperature and capnography monitoring, and early postoperative laboratory surveillance (potassium and CK), aligning with established recommendations and pathophysiological considerations in LGMD [2-4]. The uneventful course and prompt recovery in our case are concordant with prior regional-anaesthesia reports across obstetric and abdominal procedures in LGMD, while TIVA-based general anaesthesia remains a viable fallback when regional techniques are contraindicated or unsuccessful [6-12].

Pragmatic approach

For LGMD patients presenting for urgent gynaecological surgery, a practical strategy is to begin with comprehensive preoperative assessment (phenotype/subtype if known, respiratory and cardiac evaluation, baseline electrolytes and CK) [2,5]; default to neuraxial anaesthesia when not contraindicated (Table 3) and when it meets surgical requirements [2,5,7-12]; if regional is unsuitable or fails, proceed with TIVA-based general anaesthesia using minimal or no neuromuscular blockade (with quantitative monitoring if NMBs are employed) and strict avoidance of volatile agents and succinylcholine [6]; and throughout, maintain tight temperature control and end-tidal carbon dioxide (EtCO₂) monitoring, ensure immediate access to dantrolene, and obtain early postoperative potassium and CK checks in higher-risk phenotypes or after longer procedures (Table 4) [3,5].

**Table 4:** Absolute and relative contraindications to neuraxial anaesthesia ASRA: American Society of Regional Anesthesia and Pain Medicine; ESAIC: European Society of Anaesthesiology and Intensive Care; INR: International normalized ratio; MS: Multiple scleroris; ICP: Intracranial pressure; IIH: Idiopathic intracranial hypertension; GA: General anaesthesia

Absolute contraindications	Relative/conditional contraindications
Patient refusal/inability to give consent	Anticoagulation or antiplatelet therapy (agent-specific timing; follow local ASRA/ESAIC policy)
Infection at puncture site (cellulitis/abscess)	Systemic sepsis/bacteraemia remote from the site (after antibiotics/risk–benefit)
True allergy to planned local anaesthetic or additive (e.g., intrathecal opioid, chlorhexidine)	Coagulopathy or thrombocytopenia not meeting local thresholds (platelet/INR-based)
Raised ICP due to mass/space-occupying lesion with herniation risk	Pre-existing neurological disease (e.g., neuropathy, MS)—document baseline, counsel risk
Uncorrected hypovolaemia/shock	Severe fixed-output cardiac lesions (e.g., critical aortic stenosis), severe pulmonary hypertension
Severe, active bleeding/consumptive coagulopathy (until corrected)	Inability to cooperate or maintain position; significant anxiety (may improve with support/sedation)
	Significant spinal deformity, prior spine surgery/hardware (technical difficulty, altered spread)
	Anticipated prolonged surgery beyond block duration/high chance of conversion to GA
	Raised ICP not due to mass (e.g., IIH)—individualise with specialist input
	Autonomic dysfunction or unstable haemodynamics requiring close titration
	Anticipated difficult airway where immediate GA backup is essential (plan accordingly)
	Pregnancy-specific factors (e.g., refractory coagulopathy, severe pre-eclampsia with low platelets)

**Table 5:** Anaesthetic management in LGMD: case characteristics, techniques, and outcomes AD/AR: Autosomal dominant/recessive; BIS: Bispectral index; CBC: Complete blood count; CK: Creatine kinase; CSE: Combined spinal-epidural; DCM: Dilated cardiomyopathy; ECG: Electrocardiogram; ENT: Ear, nose & throat; ERPC: Evacuation of retained products of conception; EtCO₂: End-tidal carbon dioxide; GA: General anaesthesia; K⁺: Potassium; LFT/RFT: Liver/renal function tests; LGMD: Limb-girdle muscular dystrophy; MH: Malignant hyperthermia; NIBP: Non-invasive blood pressure; NIPPV: Non-invasive positive-pressure ventilation; NMB: Neuromuscular blocker; NR: Not reported; SCP: Superficial cervical plexus; SLN: Superior laryngeal nerve; sux: Succinylcholine; TCI: Target-controlled infusion; TIVA: Total intravenous anaesthesia; U&E: Urea & electrolytes

Study	Age/Gender	LGMD type	Complication	Operative procedure	Preoperative examinations	Anaesthesia method	Anaesthesia machine preparation	Induction/maintenance	Intraoperative monitoring	Postoperative pain management
Current case	31 / F	Unspecified	None	Emergency ERPC	CBC, U&E incl. K⁺, CK, LFT/RFT (normal)	Single-shot spinal	Vapour-free machine; trigger-free pathway; TIVA backup ready	0.5 % hyperbaric bupivacaine 10 mg; no sedatives / NMBs	ECG, NIBP, SpO₂, EtCO₂, temperature	Paracetamol ± NSAID
Devkota et al. [8]	NR/F	Unspecified	None	Total abdominal hysterectomy	NR	Regional (neuraxial)	Trigger-free setup	NR	Standard haemodynamic & respiratory monitoring	NR
Allen & Maguire [9]	28/F	AR-LGMD (restrictive lungs)	Respiratory risk	Emergency caesarean delivery	Respiratory assessment performed	CSE	Trigger-avoidance stated	Epidural top-ups (NR specifics)	Standard + respiratory backup ready	NR
Soni et al. [10]	NR/F	Unspecified	None	Caesarean section	NR	Single-shot spinal	Trigger-avoidance stated	NR	Standard	NR
von Guionneau et al. [11]	20/F	LGMD 2I + DCM	Cardiac risk	Pregnancy management / delivery	Cardiology input + echocardiography	Neuraxial favoured	Trigger-avoidance stated	NR	Cardio-respiratory surveillance	NR
Black & Said [12]	NR/F	AR-LGMD	Anticipated respiratory weakness	Vaginal delivery	NR	Neuraxial feasible	Trigger-avoidance stated	NR	Careful monitoring	NR
da Cunha Leal et al. [13]	NR/F	LGMD 2A	None	Abdominoplasty ± liposuction	NR	CSE	Trigger-free setup	Graded block (NR drugs)	Standard haemodynamic vigilance	NR
Cao et al. [6]	NR/F	LGMD 2B	Rhabdomyolysis risk	Non-gynaecologic surgery	NR	GA (propofol-based TIVA)	Vapourisers removed; trigger-free setup	Propofol infusion; no sux; avoid NMBs	Standard + quantitative NMB monitoring	Multimodal analgesia (NR specifics)
Ranjan et al. [14]	27/F	Unspecified	Obesity	Elective caesarean section	NR	Epidural + NIPPV	Trigger-free setup	Regional only	Standard	NR
Kulkarni et al. [15]	NR/F	Unspecified	None	Caesarean section	NR	Single-shot spinal	Trigger-avoidance noted	Regional only	Standard	NR
Yılmaz et al. [16]	NR/F	Unspecified	None	Caesarean section	NR	CSE	Trigger-free setup	Regional only	Standard	NR
Sarkılar et al. [17]	Child/M	Unspecified	None	Paediatric non-gynaecologic surgery	NR	GA (IV rocuronium + sugammadex)	Trigger-free IV agents	Propofol-based GA	ECG, SpO₂, EtCO₂	NR
Baldeón-Chávez et al. [18]	57/F; 52/M	LGMD 2A / unspecified	None	Thyroidectomy/ENT surgery	Labs, CXR, ECG	GA (TIVA with TCI)	OR per MH protocol; vapour-free	Thiopental / propofol + remifentanil (TCI)	ECG, SpO₂, EtCO₂, NIBP, temp, BIS monitoring	Morphine + non-opioids (pt 1)
López-Álvarez et al. [19]	NR/F	Unspecified	None	Ascending aortic aneurysm repair	NR	GA (propofol / remifentanil TCI)	Trigger-free setup	Rocuronium → propofol 3–5 µg/mL; remifentanil 2–3 ng/mL	Standard + controlled ventilation	NR
Shakya & Shrestha [20]	NR/F	Unspecified	None	Laparoscopic cholecystectomy	NR	GA (TIVA; no NMB)	Trigger-free setup	Propofol + dexmedetomidine	Standard	NR
Kabade et al. [21]	40/F	AD-LGMD	None	Total thyroidectomy	ECG, echo, labs	Regional blocks (bilateral SCP + SLN) + light sedation	OR per MH protocol	Midazolam 1 mg → propofol infusion + fentanyl 50 µg (O₂ mask)	Standard + temperature monitoring	Non-opioid analgesia; uneventful

Evidence remains largely case-based due to rarity and heterogeneity. Our experience contributes a detailed, trigger-free neuraxial pathway for emergency ERPC in LGMD, and emphasises the importance of postoperative biochemical monitoring particularly serum potassium and CK tailored to myopathy-related risk.

## Conclusions

In LGMD, peri-operative risk hinges on susceptibility to anaesthesia-related rhabdomyolysis, hyperkalaemia, and unpredictable responses to NMBs. For urgent gynaecological procedures such as ERPC, a trigger-free neuraxial strategy can provide rapid, reliable surgical anaesthesia while preserving spontaneous ventilation and minimising haemodynamic disturbance. In our patient, single-shot spinal anaesthesia within a rigorously prepared pathway vapour-free machine, strict avoidance of succinylcholine and volatile agents, continuous temperature and capnography monitoring, a ready TIVA contingency, and early postoperative potassium/CK checks resulted in an uncomplicated course and same-day recovery. Although evidence remains largely case-based, this report supports neuraxial anaesthesia as first-line for appropriately selected LGMD patients presenting for urgent gynaecological surgery, with propofol-based TIVA reserved for contraindications to regional techniques. Prospective data and standardised reporting would help refine risk stratification and peri-operative protocols in this population.
